# Impaired Prefronto-Thalamic Functional Connectivity as a Key Feature of Treatment-Resistant Depression: A Combined MEG, PET and rTMS Study

**DOI:** 10.1371/journal.pone.0070089

**Published:** 2013-08-02

**Authors:** Cheng-Ta Li, Li-Fen Chen, Pei-Chi Tu, Shyh-Jen Wang, Mu-Hong Chen, Tung-Ping Su, Jen-Chuen Hsieh

**Affiliations:** 1 Department of Psychiatry, Taipei Veterans General Hospital, Taipei, Taiwan; 2 Institute of Brain Science, National Yang-Ming University, Taipei, Taiwan; 3 Division of Psychiatry, Faculty of Medicine, National Yang-Ming University, Taipei, Taiwan; 4 Integrated Brain Research Unit, Department of Medical Research and Education, Taipei Veterans General Hospital, Taipei, Taiwan; 5 Center of Neuropsychiatric Research, National Health and Research Institute, ChuNan, Taiwan; 6 Department of Nuclear Medicine, Taipei Veterans General Hospital, Taipei, Taiwan; Charité University Medicine Berlin, Germany

## Abstract

Prefrontal left-right functional imbalance and disrupted prefronto-thalamic circuitry are plausible mechanisms for treatment-resistant depression (TRD). Add-on repetitive transcranial magnetic stimulation (rTMS), effective in treating antidepressant-refractory TRD, was administered to verify the core mechanisms underlying the refractoriness to antidepressants. Thirty TRD patients received a 2-week course of 10-Hz rTMS to the left dorsolateral prefrontal cortex (DLPFC). Depression scores were evaluated at baseline (W0), and the ends of weeks 1, 2, and 14 (W14). Responders were defined as those who showed an objective improvement in depression scores ≥50% after rTMS. Left-right frontal alpha asymmetry (FAA) was measured by magnetoencephalography at each time point as a proxy for left-right functional imbalance. Prefronto-thalamic connections at W0 and W14 were assessed by studying couplings between prefrontal alpha waves and thalamic glucose metabolism (PWTMC, reflecting intact thalamo-prefrontal connectivity). A group of healthy control subjects received magnetoencephalography at W0 (N = 50) to study whether FAA could have a diagnostic value for TRD, or received both magnetoencephalography and positron-emission-tomography at W0 (N = 10) to confirm the existence of PWTMC in the depression-free state. We found that FAA changes cannot differentiate between TRD and healthy subjects or between responders and non-responders. No PWTMC were found in the TRD group at W0, whereas restitution of the PWTMC was demonstrated only in the sustained responders at W14 and euthymic healthy controls. In conclusion, we affirmed impaired prefronto-thalamic functional connections, but not frontal functional imbalance, as a core deficit in TRD.

## Introduction

With its chronic and recurrent nature, major depressive disorder (MDD) is a debilitating illness of immense socioeconomic cost and is associated with high suicidality and medical comorbidity [1], [2]. Even with a combination of antidepressant drugs and psychotherapy as a treatment option, more than 30% of depressed patients are refractory to adequate trials of antidepressant treatment [3], [4]. The central mechanisms underlying treatment-resistant depression (TRD) are unclear.

Some patients with TRD can nonetheless manifest good responses to repetitive transcranial magnetic stimulation (rTMS) treatment [5]–[8], particularly when a combination of daily prefrontal rTMS and medication treatment was applied for weeks [8]. Hypoexcitability over the left prefrontal cortex (PFC) and hyperexcitability over the right PFC are commonly-accepted mechanisms to be associated with depression [5], which could explain why the initial use of the excitatory rTMS (e.g., 10 Hz) applied to the left PFC and the inhibitory rTMS (e.g., 1 Hz) to the right PFC in TRD [5]. A pattern of PFC asymmetry with relatively right predominance [e.g., frontal alpha asymmetry (FAA) at a resting state] has been proposed as an endophenotype for depression [9], [10]. A growing number of evidence also supported that both left excitatory and right inhibitory rTMS have antidepressant efficacy for TRD [5]–[7], supporting the impaired left-right PFC functional balance as one pathological attribute of antidepressant refractoriness in MDD. However, little research so far specifically investigated this issue and almost all studies on FAA in depression adopted electroencephalography, which measures electrical activity from the scalp and is limited by low sensitivity.

The PFC-thalamo-limbic (e.g., amygdala) pathway is one of the key circuits implicated in depression [11] and its disruption may be specific to refractory MDD. High-frequency prefrontal rTMS has been reported to enhance cortical excitability/activity [5], [12], [13] and to improve PFC inhibitory functions through enhanced GABA-mediated inhibitory neurotransmission [14], and therefore normalizes the PFC-limbic dys-regulation of the depressive circuit [7], [15]. The amygdala is a major structure in the emotional limbic system of human subjects with depression [16]. In the PFC-thalamo-amygdala circuit, the thalamus (e.g., the mediodorsal nucleus) is the principal relay region for PFC subregions [17] and has extensive reciprocal connections with the amygdala [18]. The functional disruption between PFC and thalamus had been found in refractory MDD [19]; however, it is unknown whether such disruption is the core pathophysiology of TRD.

To investigate the mechanism underlying antidepressant treatment refractoriness, functional neuroimaging in combination with left PFC rTMS as an intervention maneuver was performed with patients with TRD. First, in order to study left-right PFC functional imbalance, we utilized magnetoencephalography (MEG) and investigated the FAA. FAA as investigated by MEG has been demonstrated as a proxy for left-right PFC functional imbalance [20]. The magnitude of alpha power is inversely correlated with regional cortical activity in depression [21], [22]. Second, in order to verify whether the PFC-thalamic circuit was disrupted in TRD, we investigated the couplings between thalamic metabolism by ^18^F-FDG-PET and the PFC alpha activity by MEG. The presence of such PFC brain wave-thalamic metabolism coupling (PWTMC) has been proposed as a monitor for an intact thalamo-cortical connection and has been repeatedly reported to be found in healthy subjects [23]–[25]. A lack of correlation between frontal cortical alpha activity and thalamic glucose metabolism has been reported in depressed patients [25]. Our goal was to verify the core mechanisms underlying the refractoriness to antidepressants in TRD.

## Materials and Methods

### Study Subjects

Thirty TRD patients (age range, 36–63 years) without history of substance abuse and major physical or neurological illness were recruited (Table 1). A structured history was taken and the diagnosis of MDD was confirmed by administration of the Mini International Neuropsychiatric Interview (MINI), based on the *Diagnostic and Statistical Manual for Mental Disorders (DSM-IV)* criteria (American Psychiatric Association, 1994). TRD patients were defined as MDD with a confirmed history of antidepressant failures and with depression scores ≥18 (i.e., moderate-to-severe in current depression severity at entry) on the 17-item Hamilton Depression Rating Scale (HDRS-17) [26] before rTMS treatment. In order to confirm a history of antidepressant refractoriness, we recruited MDD patients who had failed to respond to at least 2 different classes of adequate antidepressant trials [27]. Patients were recruited only if they had no comorbidity with bipolar disorders, major psychoses, obsessive-compulsive disorders, post-traumatic stress disorder, or cluster B personality disorders. Highly treatment-refractory MDD was not our target (e.g., no response to electroconvulsive therapy), since we aimed to have a subgroup of TRD patients who could have good responses to the subsequent rTMS treatment and patients ever received electroconvulsive therapy might have brain functional abnormality that is not associated with the depression itself but with the electroconvulsive therapy.

**Table 1:** Demographic data and clinical variables between rTMS responders and non-responders.

	rTMS responders	rTMS non-responders	t or χ^2^	P
Gender (Female, %)	70.6%	92.3%	2.172	0.196
Age (y/o)	51.9 (10.5)	50.1 (6.2)	0.549	0.588
Education (years)	11.9 (3.5)	11.9 (3.9)	0.013	0.184
Age at onset (y/o)	41.6 (13.8)	38.9 (10.1)	0.585	0.563
Duration illness (years)	10.2 (7.7)	11.2 (9.1)	−0.315	0.755
Current MDE duration (months)	6.4 (6.5)	14.0 (29.0)	−1.052	0.302
Number of MDE episodes (times)	6.4 (4.0)	5.1 (3.5)	0.965	0.343
Baseline HDRS-17	22.6 (2.8)	23.4 (3.0)	−0.621	0.540
2-week HDRS-17	7.7 (3.0)^#^	19.1 (4.4)	−8.395	<0.001**
14-week HDRS-17	6.9 (5.2)^#^	18.9 (6.9)	−4.225	<0.001**
Baseline BDI	29.5 (8.5)	33.0 (9.4)	−1.075	0.291
2-week BDI	13.1 (7.2)^#^	30.2 (12.1)	−4.827	<0.001**
14-week BDI	13.6 (9.5)^#^	26.7 (12.0)	−3.339	0.002**

Note: MDE, major depressive episode; HDRS-17, 17-item Hamilton depression rating scales; BDI, Beck depression index; **^#^** Significant decreases (pair-*t* test, p<0.05) as compared to baseline values; *p<0.05, **p<0.005.

The study was performed in accordance with the Declaration of Helsinki and was approved by the local Ethics Review Committee (the Institutional Review Board of the Taipei Veterans General Hospital). All participants provided written informed consent.

### PET, MRI, and MEG Procedures

#### Positron emission tomography (PET)

All recruited TRD patients received two resting ^18^F-FDG PET scans with a PET/CT scanner (Discovery VCT; GE Healthcare, USA): a baseline scan before rTMS and another 3 months after the 2-week add-on rTMS treatment. Patients fasted for at least 4 h before the PET examination. A 15-minute series of PET images was acquired 45 minutes after an intravenous injection of about 370 MBq of ^18^F-FDG. The resting brain inter-regional correlations between eyes-open and eyes-closed conditions (during the 15-minute scan) were expected to be similar because the glucose metabolic patterns were determined mainly during the 45-minute resting period after the injection of ^18^F-FDG while the patient was waiting in a quiet and dimly-lighted room. To minimize the influence of a patient’s excessive eye movements on resting brain metabolism, the 15-minute PET scan was done with the subject’s eyes closed. A total of 47 consecutive slices over an axial length of 15.7 cm was obtained (slice thickness = 3.75 mm, transaxial FOV = 70 cm). A 128×128 matrix was constructed for PET images, and CT information was used to correct for attenuation [12].

#### Magnetic resonance image (MRI)

To accurately target the rTMS coil, T1-weighted MRIs (TR = 8.548 ms, TE = 1.836 ms, TI = 400 ms, flip angle = 15°, FOV = 26 × 26 cm, matrix size = 256 × 256, 124 contiguous slices and slice thickness = 1.5 mm) were done using a 1.5T MRI system (Excite II; GE Medical Systems, Milwaukee, WS, USA) before the first rTMS session for all patients.

#### Magnetoencephalography (MEG)

Three-minute MEG recordings at baseline (W0), the end of the 1^st^week (W1), the 2^nd^ week after rTMS (W2) and the 12^th^ week after the 2-week rTMS (W14) were recorded with a whole-head 306-channelneuromagnetometer (Vectoview, ElektaNeuromag, Helsinki, Finland) in resting and eyes-open states. We chose to record MEG signals during the eyes-open resting state to minimize occipital alpha’s influence on prefrontal alpha oscillation and prefronto-thalamic circuits, since occipital alpha would be significantly enhanced if the eyes were closed. Subjects were also asked to look at a fixed point on a screen just in front of them to minimize mental wandering during the resting condition. The magnetic signals were then digitized at a 1000-Hz sampling rate and filtered with a bandwidth of 0.03–300 Hz. Two additional electro-oculogram electrodes for monitoring eye movements were placed horizontally and vertically. To study whether FAA could have a diagnostic value for TRD, a group of age-, gender- and handedness-matched healthy controls (N = 50, male/female = 14/36, mean (SD) age = 49.1 (7.0) years) also underwent MEG recordings.

### rTMS Procedures

The detailed rTMS procedures were done largely as described previously [12]. To summarize, brain-navigation computer software and an infrared system were used to accurately guide the figure-8-shaped coil to target the left dorsolateral PFC (DLPFC), based on each patient’s brain MRI. Ten daily rTMS treatment sessions were administered for 2 consecutive weeks (parameters: 10-Hz, 100% motor threshold, 4 sec on and 26 sec off, 40 times/session, and 5 sessions/week). The parameters of stimulation we selected here were in accordance with the suggested safety guidelines for conventional rTMS application in human research [28] and the treatment duration of two weeks had been proved to be effective in treating refractory MDD [8], [12].

During the entire rTMS study, all patients continued their current antidepressant medications and no medication changes were allowed for at least 4 weeks preceding rTMS, and throughout the period of rTMS treatment and the 3-month follow-up; however, if depressed patients failed to respond to the 2-week add-on rTMS (rTMS non-responders) or the rTMS responders had a severe exacerbation of depressive symptoms, then changes in medications were permitted based on the clinician’s judgment. During the course of rTMS treatment, patients were using the following medications: a selective serotonin reuptake inhibitor (fluoxetine [N = 2], sertraline [N = 1], escitalopram [N = 3]) or a serotonin-norepinephrine reuptake inhibitor (venlafaxine [N = 7], duloxetine [N = 6]), a norepinephrine dopamine reuptake inhibitor (bupropion [N = 8]) or a noradrenergic/specific serotonergic agent (mirtazapine [N = 8]). In order to study the core mechanisms of TRD, we adopted add-on rTMS to vigorously treat depressive symptoms in the TRD patients; however, in order to minimize the effects of the drugs on brain activity, all patients during rTMS treatment (i.e., W0 to W2) and the rTMS responders after the rTMS treatment (W2 toW14) were kept on their original drugs.

### Patient Assessments

The Beck Depression Inventory (BDI) and HDRS-17 were utilized to assess the subjective and objective severity of depression at W0, W1, W2 and W14. The patients were further subcategorized into responder and non-responder groups, according to their clinical responses to the 2-week add-on rTMS treatment. Responders were defined by an objective improvement in HDRS-17 scores ≥50%. None of the responders had a medication change throughout the study period in order to control potential confounding effects from the drugs.

### Image Data Treatment and Analyses

#### Positron emission tomography (PET)

PET data were analyzed using Statistical Parametric Mapping version 5 software (SPM5; Wellcome Department of Cognitive Neurology, Institute of Neurology, University College London, London, England) implemented in Matlab 7.0 (The Mathworks Inc., Sherborn, MA, USA). First, an ^18^F-FDG template was created from all patients via the co-registration of each individual’s MRI. Then, a study-specific ^18^F-FDG template was used to normalize each subject’s MRI images, followed by smoothing with a 3D Gaussian kernel (FWHM = 12 mm). Global variance across the scans was removed by analysis of covariance (ANCOVA) [29]. A paired-*t* test for ^18^F-FDG uptake between W0 and W14 was done and the significance thresholds were set at a cluster-level FWE(family-wise error rates)-corrected P<0.001.

#### Magneto-encephalography (MEG)

Artifact rejection for blinks and excessive eye movements was performed in the acquired MEG signals. Epochs containing amplitudes over 6000 fT/cm were also rejected. Amplitude spectrum density analysis by Fast Fourier Transform (FFT) (4096 points, Hanning window with 50% overlap) was done for the artifact-free epochs. While the alpha band (8–13 Hz) was our primary interest, other frequency bands, including delta (2–4 Hz), theta (4–8 Hz), beta (13–30 Hz) and gamma (30–50 Hz), were also analyzed. Absolute amplitude spectra of the frontal area were calculated by averaging MEG activity from 26 gradiometers in the frontal area. The relative amplitude index for each frequency band was reported [relative amplitude index of alpha band (rAlpha) = (absolute amplitude of alpha band)/(average absolute amplitude of all 5 bands)] [30].

#### FAA as a monitor for left-right PFC functional imbalance

**Figure 1:**
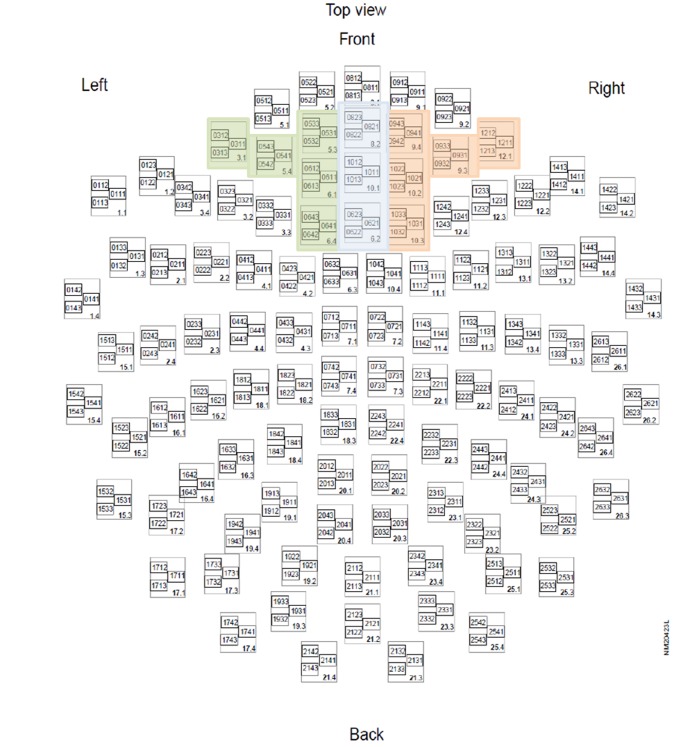
Selection of frontal sensors (all colored squares). Green: left frontal sensors; Orange: right frontal sensors.

MEG-FAA is a reliable monitor for left-right PFC functional imbalance [20]. FAA was calculated with the following equation [FAA = (left alpha activity - right alpha activity)/(left alpha activity+right alpha activity)]. Left and right frontal alpha activities were averaged from absolute alpha amplitudes of 5 pairs of gradiometers of the selected sensors from the left and right frontal areas, respectively (Figure 1). We used root-mean-squares (RMS) to combine alpha values of 2 gradiometers at any selected sensor (i.e., one pair of gradiometers in an orthogonal direction) with the following equation: RMS = Sqrt (sum(G_1_^2^+ G_2_^2^)/2). While a negative relationship between alpha activity and frontal cortical neuronal activity has been proposed [21], [22], [31], a positive value of FAA denotes a relatively lower left(versus right)PFC activity and vice versa.

#### MEG/PET correlations for prefronto-thalamic connectivity: PWTMC

Voxel-based partial correlations were performed to investigate the associations between frontal rAlpha and ^18^F-FDG glucose metabolism at W0 and W14 after factoring for age, gender and total gray matter count. Despite the presence of the PWTMC has been repeatedly reported to be found in healthy subjects [23]–[25], we also recruited a group of healthy subjects (N = 10, male/female = 2/8, mean (SD) age = 45.2 (9.8) years, HDRS-17 = 0.7 (1.1), and BDI = 1.0 (1.7)) to confirm a correlation between frontal alpha oscillation (MEG) and thalamic GluM (PET) at W0. A cluster-level P<0.001 (FWE-corrected for multiple comparisons) was thought to be significant. Since the presence or absence of PWTMC under the statistical threshold set for tests of significance in each group (e.g., TRD and healthy controls) or each condition (e.g., TRD at W0 and at W14) is an all-or-none phenomenon, Z transform is deemed unnecessary here.

### Statistical Analyses for Demographic Data, Clinical Characteristics and Brain Neuromagnetic Variables

Student’s *t* test and Fisher’s chi-square test were used to compare the continuous and categorical variables between groups, respectively. The paired-*t* test was used to compare continuous variables within groups. After controlling for age and gender, the partial correlation test was used for relationships between baseline band activity and severity of depression (HDRS-17 scores) and changes in HDRS-17 scores before and after rTMS. P<0.05 was deemed statistically significant.

## Results

### Demographic Data and Clinical Characteristics

The number of failed adequate antidepressant trials that our TRD patients experienced (as confirmed by self-reports and by a review of the antidepressant prescription patterns of their medical records) ranged from four to nine, and all of the recruited TRD patients failed to respond to drug combination therapy by either antidepressant or antipsychotic combinations. All TRD patients (N = 30, male/female = 6/24) received add-on rTMS treatment. Seventeen (56.7%) responded and such response rate was similar to the results (active vs. sham rTMS = 60% vs. 10%) of the double-blind, sham-controlled add-on rTMS study that we had done in a separate group of patients with refractory depression [8]. The responders and non-responders did not differ in demographic and clinical characteristics such as age at onset, duration of illness, previous depressive episodes and medications. The responders had expectedly lower depression scores than did the non-responders on the HDRS-17 (P<0.001) and BDI (P<0.001) at the end of the 2-week rTMS course (Table 1). The antidepressant effect outlasted the entire follow-up period in the rTMS sustained responders (N = 12, out of 17 add-on rTMS responders at W2).

### Brain Neuromagnetic Variables and their Correlation with Clinical Variables

**Figure 2:**
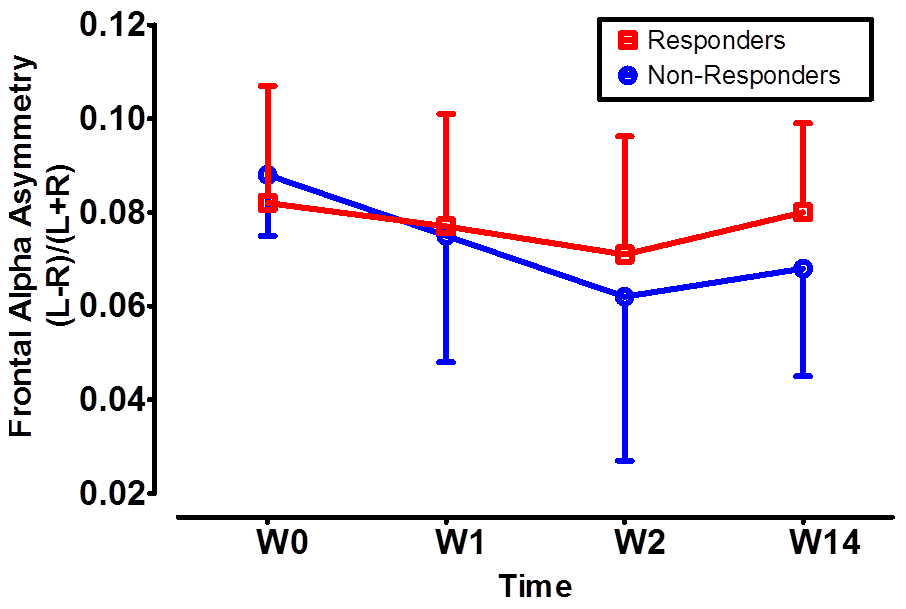
rTMS’s cumulative effects on reversing frontal alpha asymmetry (FAA). FAA of rTMS responders (in red color) and non-responders (in blue color) showed no difference from baseline (W0) to the end of the 1^st^ week (W1) and the 2^nd^ week (W2) after initiation of rTMS. In both groups, rTMS decreased FAA in a dose-dependent manner from W0 to W2, despite lack of statistical significance. During the follow-up period from the end of W2 to the 14^th^ week (W14), no active rTMS was used, and we observed a gradual rebound of FAA to its baseline value in both groups.

Baseline FAA in healthy controls and TRD patients was comparable. Both TRD groups (i.e., responders and non-responders) demonstrated a dose-dependent decrease in FAA non-significantly in response to the accumulated rTMS stimulations for the 2 weeks (Figure 2); however, a gradual rebound back to the baseline FAA level was observed during the follow-up period (W2 to W14, without active rTMS stimulations). We also checked the overall frontal alpha activity changes over time, but there was no significant finding (data not shown). No significant correlation existed between HDRS-17 scores and FAA (W0: r = 0.009, p = 0.967; W14: r = −0.106, p = 0.647) or frontal rAlpha activity (W0: r = −0.056, p = 0.800; W14: r = 0.006, p = 0.978). In addition, no statistical significance could be observed between HDRS-17 percentage changes and baseline frontal rAlpha activity (r = −0.278, p = 0.200) and baseline FAA (r = 0.295, p = 0.182).

### MEG/PET Correlation to Assess Prefronto-thalamic Connections

**Figure 3:**
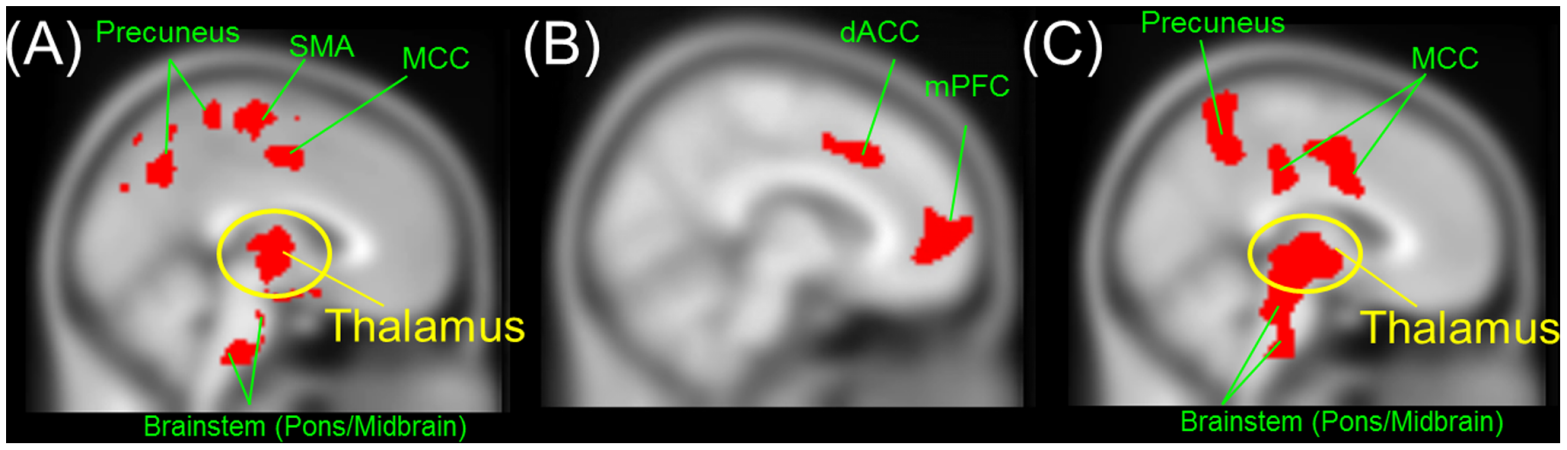
Correlations between MEG frontal alpha activity and PET glucose metabolism in healthy controls and in depression before and after successful rTMS treatment. (A) *In healthy subjects*. Frontal alpha activity correlated well with glucose metabolism in the thalamus (circled in yellow). (B) *Before rTMS.* Frontal alpha activity correlated well with glucose metabolism in various parts of the prefrontal cortex, but did not correlate with thalamic activity. (C) *After successful rTMS treatment*. Frontal alpha activity correlated well with glucose metabolism in the thalamus (circled in yellow), brainstem, precuneus, and cingulate cortices. mPFC, medial prefrontal cortex; dACC, dorsal anterior cingulate cortex; MCC, middle cingulate cortex; SMA, supplementary motor area. Brain regions showing significant negative correlations (cluster-level corrected P<0.001) in each condition are shown in red color.

Prefrontal alpha wave and thalamic glucose metabolism coupling (PWTMC) was observed in the healthy normal subjects (Figure 3A and Figure S1A). The normal pattern of PWTMC was not present in the TRD at baseline (Figure 3B), yet TRD patients demonstrated significant correlations between frontal rAlpha activity and brain glucose metabolism in various parts of the frontal cortex (corrected P<0.001, Figure S1B). After successful treatment with rTMS, patients who maintained symptomatic relief at W14 restored the PWTMC pattern (Figure 3C and Figure S1C). It is noteworthy that the loss of the PWTMC persisted at W14 in the non-responders (Figure S3), even though most of the rTMS non-responders were treated more aggressively after W2 with other kinds of antidepressants (i.e., 76.9% of the patients changed their antidepressant regimens, of these, 70% took antidepressant combinations and 30% took monotherapy with another kind of antidepressants). These MEG/PET findings supported the premise that PFC-thalamic disruption was critically involved in the central mechanism of TRD.

**Figure 4:**
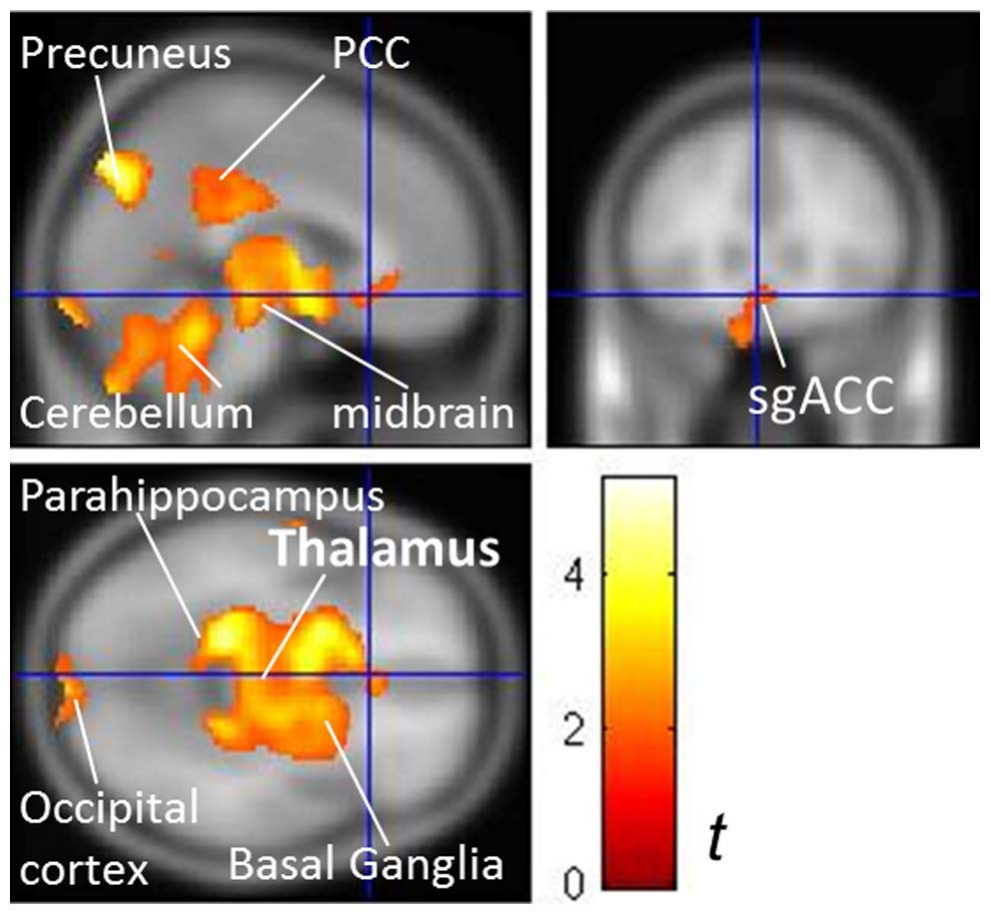
rTMS-related metabolic change in responders (*3-month vs. baseline*). Responders demonstrated significantly decreased metabolism in the thalamus, midbrain, cerebellum, posterior cingulate cortex (PCC), basal ganglia, occipital cortex, parahippocampus and subgenual anterior cingulate cortex (sgACC). Contrast bar denotes *t* values. The significance was set at a cluster-level corrected P<0.001 by paired-*t* tests.

Besides, only in the responders did the after-versus-before brain glucose metabolic changes show a decrease in the thalamus, cerebellum and limbic structures like the sgACC, caudate/putamen, precuneus and posterior cingulate cortex (cluster-level, corrected P<0.001) (Figure 4). In the non-responders, there were no significant metabolic decreases (uncorrected P<0.05, Figure S2).

## Discussion

In this first study combining MEG, PET and MRI-navigated rTMS in the investigation of TRD, we affirmed an impaired PFC-thalamus reciprocal connection, but not PFC left-right functional imbalance, as a core functional deficit in TRD pathophysiology. The evidence for this was a recoupling of PWTMC (by MEG/PET) in the sustained responders. Our results suggested that an intact PFC-thalamic functional network was an important compensatory pathway for TRD patients to control their depressed mood.

PWTMC could serve as a monitor for an intact cortico-thalamo-cortico connection [23]–[25], [32]. The loss of such coupling might thus reflect a disconnection between the PFC and the thalamus in TRD. The integrity of PWTMC is essential for a healthy life and is related to the euthymic state in MDD, as reflected by the presence of the PWTMC couplings in both the healthy subjects and the TRD sustained responders. The loss of PWTMC implied that thalamic functional abnormality could be a cause for the observed PFC-thalamus functional disruption. Alpha oscillation is generated from the thalamic reticular nucleus and such coupling remained observable even if animals were decorticated with an intact thalamus [32]. If the thalamus is abnormally depolarized or hyperpolarized, however, such neurophysiological coupling would disappear. Consistent with our findings, frontal alpha/thalamic metabolic coupling in healthy subjects and a loss of coupling in depressed patients has been reported [25]. In addition, functional aberrancy and even structural abnormalities in the thalamus have been reported to be associated with refractory depression [33], [34]. A remission in major depression was associated with significant decreases in bilateral thalamic activity [35], and lower pretreatment activity in the thalamus was related to a better response to antidepressant treatment [36].

Hypoexcitability of the frontal cortex and associated GABA(A) receptor-mediated inhibitory deficits had been suggested as specific to refractory depression [33]. Structural atrophy in the left DLPFC has been specifically linked to resistance to antidepressants in MDD [33]. rTMS targeted at the left DLPFC could enhance its function. Although cortical alpha activity originates from the thalamus in a normal subject, cortical alpha activity tends to be less dominated by the thalamus in a thalamo-cortical dysregulated condition. We demonstrated that in the TRD subjects during depression, frontal alpha activity was negatively correlated with frontal, but not thalamic, glucose metabolism. The inverse correlation between frontal alpha activity and frontal glucose metabolism in depression was confirmed [9], [37].

Left-right PFC functional imbalance as reflected by MEG-FAA was expectedly improved some after rTMS treatment, yet this effect was common in the responders and non-responders and was not statistically significant in each group (Figure 2). The finding is in line with one recent rTMS-electroencephalography study, in which the investigators found that there was no change in the FAA following a 10-Hz rTMS treatment to the left DLPFC in a small group of MDD patients (N = 8, all achieved full remission after rTMS treatment) [38]. They therefore suggested that frontal alpha asymmetry can be considered a trait marker for depression. By using MEG in a larger sample of MDD patients, we confirmed their findings and expanded the findings from responders to non-responders.

Instead, thalamic suppression and PWTMC occurred only in the sustained responders. These findings suggested that a functional normalization in the thalamus and in the thalamo-PFC bottom-up network could be more specifically implicated in the core central mechanism underlying TRD. In fact, perturbation in thalamo-cortical resonance [i.e., thalamo-cortical dysrhythmia (TCD)] has been proposed to underlie a variety of neuropsychiatric disorders including depression [39]. rTMS in combination of electroencephalography has been proposed as a platform to study potential therapeutic efficacy and underlying electrophysiological mechanisms across various TCD-related neuropsychiatric disorders, since rTMS has a potential to restore proper neural oscillations [40]. A disease caused by TCD denotes a temporal relationship between thalamic aberrancy and then the occurrence of TCD and associated symptoms. Our study further confirmed that the TCD problem was involved in the pathophysiology of TRD, as evidenced by the loss of PWTMC in the TRD but the restitution of PWTMC only in the sustained responders. The failure to improve after PFC modulation in the non-responders implied that the TCD (i.e., the circuit from the thalamus to the PFC) may be even more crucial to treatment-resistant pathophysiology than the top-down depression circuit from the PFC to the thalamus.

There were some points for further consideration. First, the observed antidepressant responses could be arguably resulted from a mixture of treatment and placebo effects. However, we did not set out to investigate antidepressant mechanisms specific to rTMS treatment. Instead, we used add-on rTMS as a means to vigorously control depressive symptoms in TRD patients and studied before-versus-after brain changes. Therefore, a sham-controlled arm was not necessary. Whether rTMS could reverse the prefronto-thalamic connections after 2-weeks of treatment remains unclear and warrants further study (e.g., a sham-controlled study comparing baseline and 2-week PET in the drug-free responders). Second, gender differences on the findings were not investigated, since most TRD patients recruited in the current study were female. Third, whether the prefronto-thalamic network problem is the only mechanism for TRD and whether it is also involved in central mechanisms for highly treatment-refractory depression (e.g., no responses to electroconvulsive therapy) warrant further research to confirm.

## Conclusions

Impaired prefronto-thalamic functional connections, but not frontal left-right functional imbalance, are the core deficit for antidepressant refractoriness in TRD. Our results suggested an intact prefronto-thalamic network as an important compensatory mechanism for those with TRD to become euthymic.

## Supporting Information

Figure S1 **Correlations between MEG frontal alpha activity and PET glucose metabolism in healthy controls and in depression before and after successful add-on rTMS treatment.** (A) *In healthy subjects*. Frontal alpha activity correlated well with glucose metabolism in brain regions such as the thalamus (circled in yellow) as well as pons, precuneus, middle cingulate cortex, supplementary motor area, and hippocampus/parahippocampus. (B) *Before treatment.* Frontal alpha activity correlated well with glucose metabolism in various parts of the prefrontal cortex and precuneus, but did not correlate with thalamic activity. (C) *After successful treatment*. Frontal alpha activity correlated well with glucose metabolism in the thalamus (circled in yellow), as well as the brainstem, putamen, temporal, parietal, frontal and cingulate cortices. mPFC, medial prefrontal cortex; DLPFC, dorsolateral prefrontal cortex; dACC, dorsal anterior cingulate cortex; MCC, middle cingulate cortex; PCC, posterior cingulate cortex. Brain regions showing significant negative correlations (p<0.001 corrected for multiple comparisons) in each condition are shown in red color.
(TIF)

Figure S2 **Metabolic change in non-responders (** ***3-month vs. baseline)*** **.** Non-responders demonstrated a non-significant metabolic increase (uncorrected P<0.05) in the middle temporal cortex and bilateral fusiform gyri was found, but there were no significant metabolic decreases. Contrast bar denotes *t* values.
(TIF)

Figure S3 **No correlations between MEG frontal alpha activity and PET thalamic glucose metabolism in patients remaining depressed after add-on rTMS treatment, even when the threshold was lowered to a voxel-level uncorrected p<0.001.**
(TIF)
