# Supplementary figures and images for: Impaired Prefronto-Thalamic Functional Connectivity as a Key Feature of Treatment-Resistant Depression: A Combined MEG, PET and rTMS Study
Source: PLoS One. 2013 Aug 2;8(8):e70089. doi: 10.1371/journal.pone.0070089 (PMC3732278; doi:10.1371/journal.pone.0070089)

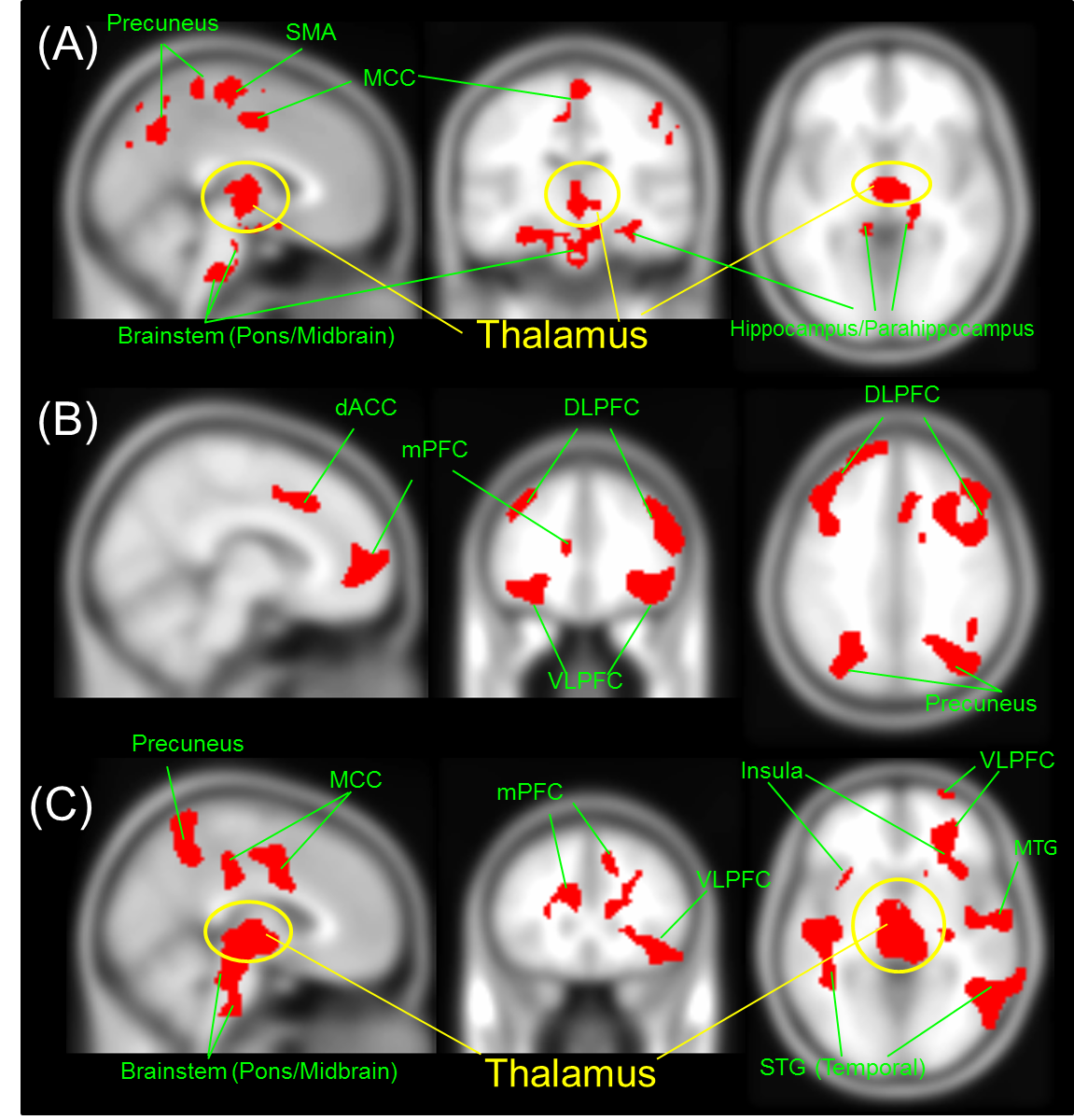

Supplement: Figure S1 — Correlations between MEG frontal alpha activity and PET glucose metabolism in healthy controls and in depression before and after successful add-on rTMS treatment. (A) In healthy subjects. Frontal alpha activity correlated well with glucose metabolism in brain regions such as the thalamus (circled in yellow) as well as pons, precuneus, middle cingulate cortex, supplementary motor area, and hippocampus/parahippocampus. (B) Before treatment. Frontal alpha activity correlated well with glucose metabolism in various parts of the prefrontal cortex and precuneus, but did not correlate with thalamic activity. (C) After successful treatment. Frontal alpha activity correlated well with glucose metabolism in the thalamus (circled in yellow), as well as the brainstem, putamen, temporal, parietal, frontal and cingulate cortices. mPFC, medial prefrontal cortex; DLPFC, dorsolateral prefrontal cortex; dACC, dorsal anterior cingulate cortex; MCC, middle cingulate cortex; PCC, posterior cingulate cortex. Brain regions showing significant negative correlations (p<0.001 corrected for multiple comparisons) in each condition are shown in red color. (TIF) [file pone.0070089.s001.tif]

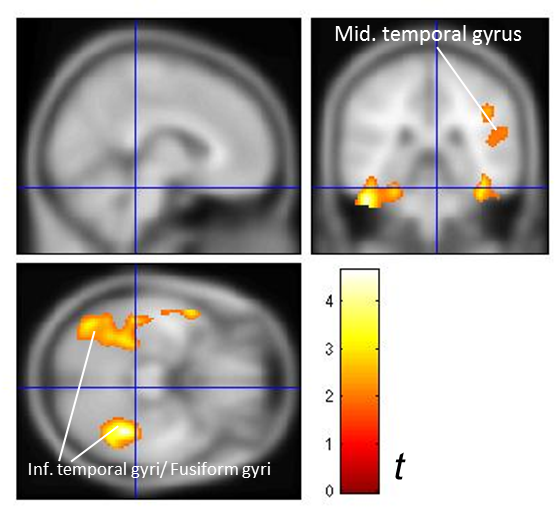

Supplement: Figure S2 — Metabolic change in non-responders ( 3-month vs. baseline) . Non-responders demonstrated a non-significant metabolic increase (uncorrected P<0.05) in the middle temporal cortex and bilateral fusiform gyri was found, but there were no significant metabolic decreases. Contrast bar denotes t values. (TIF) [file pone.0070089.s002.tif]

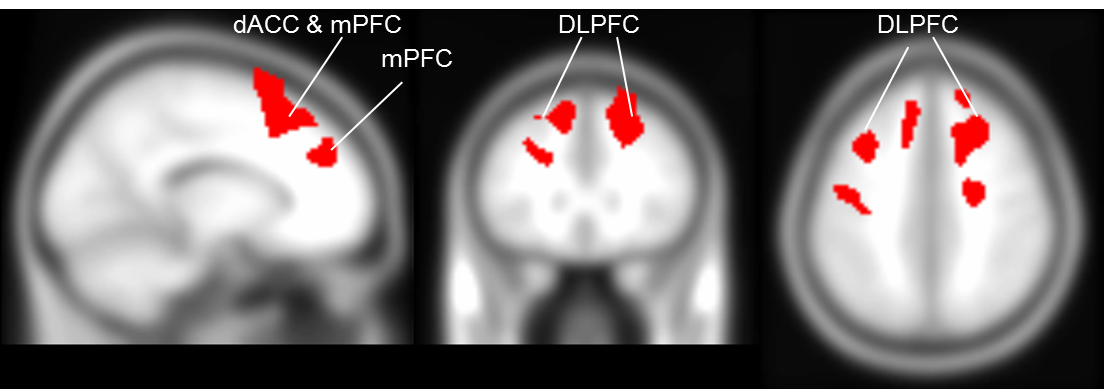

Supplement: Figure S3 — No correlations between MEG frontal alpha activity and PET thalamic glucose metabolism in patients remaining depressed after add-on rTMS treatment, even when the threshold was lowered to a voxel-level uncorrected p<0.001. (TIF) [file pone.0070089.s003.tif]
